# Clopidogrel, a Platelet P2Y12 Receptor Inhibitor, Reduces Vascular Inflammation and Angiotensin II Induced-Abdominal Aortic Aneurysm Progression

**DOI:** 10.1371/journal.pone.0051707

**Published:** 2012-12-20

**Authors:** Ou Liu, Lixin Jia, Xiaoxi Liu, Yueli Wang, Xiaolong Wang, Yanwen Qin, Jie Du, Hongjia Zhang

**Affiliations:** 1 Department of Cardiovascular Surgery, Beijing Anzhen Hospital, Capital Medical University, Beijing, China; 2 The Key Laboratory of Remodeling-related Cardiovascular Diseases, Capital Medical University, Ministry of Education; Beijing Anzhen Hospital, Capital Medical University, Beijing Institute of Heart Lung and Blood Vessel Diseases, Beijing, China; Heart Center Munich, Germany

## Abstract

Medial degeneration and inflammation are features of abdominal aortic aneurysms (AAAs). However, the early inflammatory event initiating aneurysm formation remains to be identified. Activated platelets release abundant proinflammatory cytokines and are involved in initial inflammation in various vascular diseases. We investigated the role of platelets in progression of AAA *in vivo* and *in vitro*. Histological studies of tissues of patients with AAA revealed that the number of platelets was increased in aneurysm sites along with the increased infiltration of T lymphocytes and augmented angiogenesis. In a murine model of AAA, apolipoprotein E-knockout mice infused with 1,000 ng/kg/min angiotensin II, treatment with clopidogrel, an inhibitor of platelets, significantly suppressed aneurysm formation (47% decrease, P<0.05). The clopidogrel also suppressed changes in aortic expansion, elastic lamina degradation and inflammatory cytokine expression. Moreover, the infiltration of macrophages and production of matrix metalloproteinases (MMPs) were also significantly reduced by clopidogrel treatment. *In vitro* incubation of macrophages with isolated platelets stimulated MMP activity by 45%. These results demonstrate a critical role for platelets in vascular inflammation and AAA progression.

## Introduction

Abdominal aortic aneurysm (AAA), a major disease affecting the aorta, is common among people over the age of 65 [1]. Currently, no therapy exist to prevent the development of AAA [2].

The natural history of AAA is expansion and rupture [2]. Pathological processes involve biochemical, cellular and proteolytic influences and biomechanical factors. The formation and development of AAA is characterized by aortic wall inflammation and progressive degradation of extracellular matrix proteins [3], [4]. Studies have demonstrated infiltration of inflammatory cells, such as macrophages, T cells, neutrophils [5] and dendritic cells [6], into the aortic wall. The infiltration of these inflammatory cells is considered a pathogenic mediator of AAA [7]. However, early events that initiate infiltration of the inflammatory cells into the aortic wall have not been clearly defined.

Platelets are small, regularly shaped clear cell fragments derived from precursor megakaryocytes in the bone marrow [8]. The function of platelets includes wound healing, secretion of cytokines and leukocyte interactions. Platelets facilitate clotting of blood to produce hemostasis and thrombosis and also contribute to endothelial dysfunction and modulate various inflammatory responses in vascular diseases [9], [10]. Dai et al found that platelet activation was involved in progression of AAA [11]. However, clinical studies demonstrated that anticoagulants might lead to adverse clinical events including recurrence and rupture in the treatment of aortic dissection [12], [13], [14]. Therefore, the releationship between platelets and AAA progression remain unclear [11], [12], [13], [14], [15], [16].

In this study, we explored the roles of platelets on AAA progression with both human AAA samples and a murine AAA model. We found platelet deposition in both the aorta wall of human AAA and mouse model of AAA. Clopidogrel treatment significantly prevented the progression of AAA in angiotensin II (Ang II)-infused apolipoprotein E (ApoE)-knockout mice. Inhibition of platelet substantially decreased inflammatory cells recruitment, reactive oxygen species (ROS) production and MMP activation in macrophages.

## Materials and Methods

### Patient specimens

Surgical specimens were obtained from AAA patients undergoing elective repair at Beijing Anzhen Hospital. The control aortic samples were obtained from heart transplantation donors at Beijing Anzhen Hospital. All protocols involving human specimens were approved by the Institutional Review Board at Beijing Anzhen Hospital. Each subject provided their written informed consent.

### Experimental animals

Animal experiments were conducted in accordance with experimental protocols that were approved by the Institutional Animal Care and Use Committee at Capital Medical University; all experiments conformed to the Guide for the Care and Use of Laboratory Animals published by the US National Institutes of Health (NIH Publication No. 85-23, revised 1996). ApoE- knockout mice in C57BL/6 background were from the Jackson Laboratory. After at least 48-hr acclimatization, mice (8–10 week old, male) were randomly assigned to 3 groups for treatment: infusion of normal saline (placebo control), Ang II (1,000 ng/kg/min, Sigma, St. Louis, MO), or Ang II plus clopidogrel (30 mg/kg injected intraperitoneally, Sanofi-Aventis, Tokyo, Japan). Clopidogrel, a 75-mg tablet, was dissolved in 0.9% saline under sterile conditions. Daily treatment with clopidogrel was initiated 1 week before Ang II infusion and continued throughout the study. After 4 weeks, animals were anesthetized with an intraperitoneal injection of pentobarbital (40 mg/kg). Then the abdominal and thoracic cavities were isolated, blood was drawn from the right ventricle for lipid analysis, and aortas were perfused with normal saline and fixed with 10% phosphate-buffered formalin at physiological pressure for 5 min [17]. Under a dissection microscope (SM2-1000, Nikon, Tokyo, Japan), the abdominal aortas were exposed and measured the maximal aortic diameter. The abdominal aortas (from the last intercostal artery to the ileal bifurcation) were harvested, weighed, fixed for 24 hr, embedded in paraffin, and underwent elastin or Carstairs staining or used for immunostaining.

AAA is defined as ≥50% enlargement of the maximal abdominal aorta diameter. Necropsy was performed as soon as the animals expired before sacrifice. Considering tissue degradation, these animals were excluded in the histological analysis, but used only for mortality data.

### Implantation of mini-osmotic pumps

Osmotic pumps (Alzet MODEL 2004, DURECT, Cupertino, CA) were loaded with individual concentrations of Ang II to ensure the delivery of 1,000 ng/kg/min of Ang II and inserted subcutaneously into the anesthetized mice through a small incision on the back of the neck.

### Blood pressure measurement and aortic monitoring by ultra-high frequency ultrasonography

Systolic blood pressure (SBP) was obtained 1 week before the implantation of the mini-osmotic pumps in mice and the last 3 days of the study by use of a noninvasive tail-cuff system (BP-98A, Softron, Tokyo, Japan) [18]. Aorta imaging was performed at intervals up to 28 days after mini-pump implantation by use of a high-resolution Micro-Ultrasound system (Vevo 2100, VisualSonics, Toronto, Canada) equipped with a 30-MHz transducer. Color Doppler examination was performed to detect arterial flow.

### Bleeding time

Bleeding time was assessed in mice by a tail transection method [19]. Briefly, the mouse tail was kept steady and immersed in saline thermostated at 37°C before sacrifice. After 2 min, we transected the tip of the tail with a razor blade 2 mm from the tail end. The tail was reimmersed in warm saline immediately, and the bleeding time was recorded. The end point was an arrest of bleeding lasting for more than 30 sec. The maximum bleeding time recorded was 900 sec.

### Immunohistochemistry and staining for elastin and platelets

Formaldehyde-fixed paraffin sections from mouse aortas were incubated at 4°C overnight with primary antibodies against CD41 (1∶100 dilution), CD8 (1∶200 dilution), CD31 (1∶100 dilution) or MMP2 (1∶1000 dilution; all Abcam, Cambridge, MA); monocyte chemoattractant protein 1 (MCP-1; 1∶400 dilution) or Mac2 (1∶400 dilution; both Santa Cruz Biotechnology, Santa Cruz, CA), or α-smooth muscle actin (clone 1A4, 1∶400 dilution; Sigma, St. Louis, MO). Negative controls were omission of the primary antibody, or with goat non-immune IgG, rabbit non-immune IgG or secondary antibody only; in all cases, negative controls showed insignificant staining. Immunostaining data were quantified with blinded manner to the treatment groups.

For elastin staining, tissue samples were embedded in paraffin, cut, then stained with van Gieson staining using a commercial Kit (Maixin, Fuzhou, China). Platelet staining was performed by the Carstairs staining Kit (Electron Microscopy Sciences, Hatfield, USA) according to the manufacturer's instructions.

### MMP activity

For *in situ* detection of gelatinolytic activity, mouse aortic tissues were embedded vertically in OCT (Tissue-Tek; Miles Inc., Elkhart, Illinois, USA), frozen and cut into serial 10-µm sections. Freshly cut frozen aortic sections were incubated in a dark humidified chamber at 37°C with a fluorogenic gelatin substrate (DQ gelatin, Molecular Probes, Eugene, OR) dissolved to 25 mg/ml in zymography buffer (50 mM Tris-HCL pH 7.4 and 15 mM CaCL_2_) according to the manufacturer's protocol. The sections were examined by a Nikon Eclipse TE2000-S microscope (Nikon, Japan) and analyzed by a blinded manner with use of Image Pro-Plus 3.0 (Nikon). Proteolytic activity was detected as bright green fluorescence (530 nm).

To examine the role of platelets in macrophage-derived MMP activation, mice macrophages and platelets were isolated as described below. Macrophages (1×10^5^) were plated onto 12-well plates precoated with melted gelatin solution containing DQ gelatin (50 mg/ml, Molecular Probes). Freshly isolated platelets were added at 1∶1 ratio to macrophages in half of the plates. Cells were incubated for 20 hr at 37°C. Proteolysis of DQ-gelatin (green fluorescence) was observed in live cells with use of a fluorescence inverted microscope (Leica Imaging Systems Ltd, Cambridge, UK).

### ROS analysis

Dihydroethidine hydrochloride (5 µM, Molecular Probes) was used to evaluate the *in situ* production of ROS. Freshly cut frozen mouse aortic sections were incubated with dihydroethidium (DHE; 10 µM) for 30 min at 37°C [20]. Sections were examined by Nikon Eclipse TE2000-S microscope to reveal the presence of ROS as red fluorescence (585 nm). All sections are shown with the luminal aspect facing upwards and the adventitia facing downwards.

### Preparation of mouse macrophages and platelets

Peritoneal macrophages from C57BL/6 mice were prepared as described [21]. Briefly, mice were injected intraperitoneally with 1.5 ml of 3.85% Brewer's thioglycolate solution (BD PharMingen, San Diego, CA). After 4 days, macrophages were harvested by lavage with 5 ml PBS. The cells were spun down and resuspended in fresh growth medium (DMEM) and seeded in slide chambers. Two hours after seeding, cultures were washed with PBS to remove non-adherent cells and fresh growth media was applied to establish primary cultures.

Murine platelets were prepared as described [22]. Briefly, animals were anesthetized and bled by cardiac puncture. Blood was collected into syringes containing acid citrate dextrose and spun at 100 g for 15 min. The platelet-rich plasma was obtained and spun at 1,000 g for 10 min. Washed platelets were resuspended in Tyrodes buffer (134 mM NaCl, 2.9 mM KCL, 0.34 mM Na_2_PO_4_, 12 mM NaHCO_3_, 20 mM HEPES, 1 mM MgCl_2_, 5 mM glucose and 0.5 mg/ml BSA [pH to 6.5]). To activate platelets, thrombin (0.5 U/ml) was added.

### Macrophage migration assay

Cell migration was quantitated in duplicate by use of 24-well Transwell inserts with polycarbonate filters (8-mm pore size) (Corning Costar, Acon, MA). Macrophage (2.5×10^3^ in 250 µL DMEM high-glucose medium/10% FBS) was added to the upper chamber of the insert. The lower chamber contained macrophages(1.0×10^5^) alone, activated platelets (1.0×10^8^) alone or activated platelets (1.0×10^8^) and macrophages cocultured in 1 mL RPMI 1640 medium/10% FBS isolated from WT mice. The plates were incubated at 37°C in 5% CO_2_ for 18 hr. Cells that had migrated were counted by use of DAPI staining. The cells on the top of the membrane were scrraped prior to staining. For each group, 10–20 fields were chosen randomly to count migrated macrophage and were analyzed in double-blind fashion.

### Statistical analysis

Quantitative results are expressed as mean ± SD. Differences between 2 groups were analyzed by Student's *t* test and among multiple groups by one-way ANOVA. Fisher exact test was used to analyze categorical data. Mann-Whitney test was used to analyze distribution of elastic lamina degradation grades. Data were analyzed by use of GraphPad software (GraphPad Prism version 5.00 for Windows; GraphPad Software). A P<0.05 was considered statistically significant.

## Results

### Platelets deposition in human aortas with or without AAA

**Figure 1:**
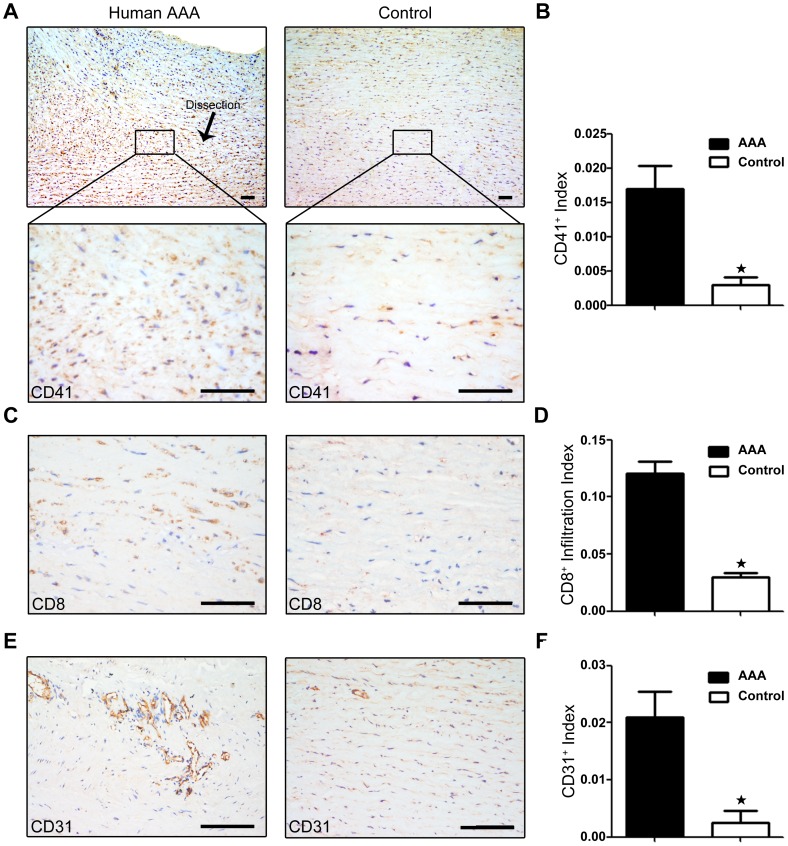
Platelet deposition in human aortas with or without abdominal aortic aneurysm (AAA). **A**, Representative CD41 immunohistochemical staining. **B**, CD41 expression in human aortas with AAA (n = 3) compared with control (n = 3). *P<0.05. **C**, Representative CD8 staining. **D**, CD8^+^ T-cell infiltration in human aortas with AAA (n = 3) compared with control (n = 3). *P<0.05. **E**, Representative immunostaining of CD31. **F**, CD31 staining of human aortas with AAA (n = 3) compared with control (n = 3). *P<0.05. Bar = 50 µm.

To establish a role for platelets in AAA progression, we evaluated the platelet deposition in human aortas with or without AAA. Immunostaining analysis demonstrated large numbers of platelets in the aortic wall of AAA lesions compared to normal aortas, as assessed by the platelet-specific marker CD41 (Fig. 1A&B). Because inflammation is involved in the progression of AAA, we then examined CD8^+^ T-cell infiltration, an early event of inflammation [23], and microvessel formation. As shown in Figures 1C&D, the number of infiltrating CD8^+^ T cells was greater in the human aortic wall of AAA lesions than in normal aortic tissue. The number of microvessels identified by CD31-positive staining was also markedly increased in the AAA lesions, consistent with the enhanced inflammatory response (Fig. 1E&F). These data established an association between the platelets deposition, inflammatory cell infiltration and AAA in patients.

### Clopidogrel prolonged bleeding time but did not affect SPB or lipid profile in mice

To determine a mechanistic link between platelet deposition and AAA formation, we inhibited platelet activities with the antiplatelet drug clopidogrel in Ang II-infused ApoE-knockout mice, a murine model of AAA. As evidence of the effectiveness of platelet inhibition, intraperitoneal injection of clopidogrel significantly prolonged bleeding time in Ang II-infused mice (Ang II only, 108.9±22.3 sec, n = 11; Ang II+ clopidogrel, >900 sec, n = 13; p<0.05, Table 1).

**Table 1:** Clopidogrel treatment prolonged bleeding time without any effect on lipid and blood pressure.

Item		Saline(n = 5)	AngII(n = 11)	AngII+Clog(n = 13)	p
Lipid fractions, mg/dL	Total cholesterol	782.3±48.6	836.2±54.1	789.9±69.7	NS
	Triglycerides	188.4±32.7	188.8±36.9	177.2±15.4	NS
Systolic blood pressure, mm Hg	Before treatment	105.7±6.2	106.3±8.3	105.5±5.9	NS
	After infusion	104.4±6.9	150.4±9.7†	149.3±10†	P<0.05
Bleeding time,second		116.8±31.6	108.9±22.3	>900*	P<0.05
AAA formation, n (%)		None	8 (66.7)	3 (20.0)*	P<0.05
AAA rupture, n (%)		None	5 (62.5)	2 (66.7)	NS

Effect of clopidogrel and angiotensin II (Ang II) on lipid levels, systolic blood pressure and bleeding time in apolipoprotein E (ApoE)-knockout mice.

† P<0.05 compared with saline infusion or before treatment.

* P<0.05 compared with control or Ang II-treated only mice.

NS, no significant difference.

Results are mean ± SD.

Ang II treatment in mice increased SBP from a baseline of 106.3±8.3 to 150.4±9.7 mm Hg after 28 days' infusion (Table 1). Intraperitoneal injection of clopidogrel did not affect mouse blood pressure. All ApoE-knockout mice showed severe hyperlipidemia. However, neither infusion of Ang II nor treatment with clopidogrel significantly affected the levels of total cholesterol or triglycerides determined at the conclusion of the study (Table 1).

### Platelet inhibition blocks Ang II-induced AAA formation

**Figure 2:**
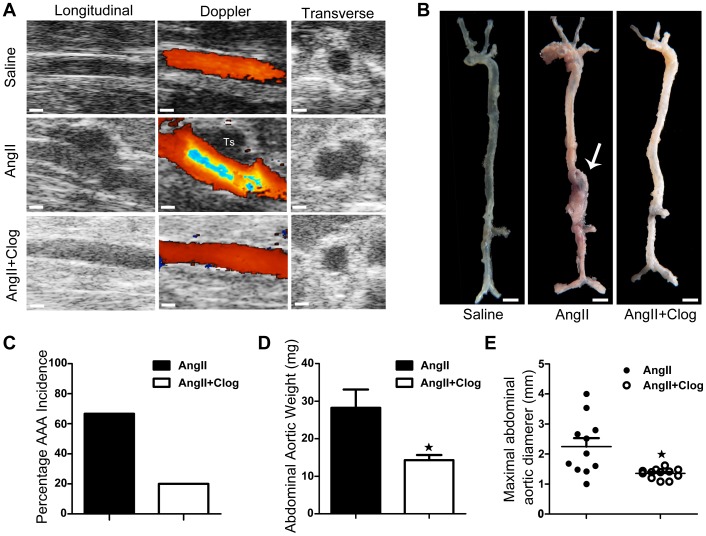
Platelet inhibition prevents angiotensin II (Ang II)-induced AAA formation in mouse aortas. **A**, Representative ultrasonography images of aortas from mice treated with saline (above), Ang II (center) and Ang II+clopidogrel (below).Ts = thrombus. **B**, Representative photographs showing macroscopic features of aortas from mice treated with saline (left), Ang II (center), and Ang II+clopidogrel (Ang II+Clog) (right). Arrows indicate typical aortic aneurysms. **C**, The incidence of Ang II-induced AAA in Ang II-treated mice (n = 12) compared with Ang II+clopidogrel-treated mice (n = 15). *P<0.05 vs. Ang II. **D**, The abdominal aortic weight of Ang II-treated mice (n = 11) compared with Ang II+clopidogrel-treated mice (n = 13). *P<0.05 vs. Ang II. **E**, Maximal abdominal aortic diameter in Ang II-treated mice (n = 11) and Ang II+clopidogrel-treated mice (n = 13). Lines represent the mean. *P<0.05 vs. Ang II.

Ultrasonography was used to measure AAA at intervals for up to 28 days after Ang II infusion (Fig. 2A). Notably, AAA formation was significantly reduced in Ang II-infused ApoE-knockout mice by clopidogrel treatment (incidence 20% vs. 67%, P<0.05, Fig. 2 B&C). In addition, effects of Ang II-infusion on the weight (Fig. 2D) and size (Fig. 2E) of aortas were significantly (P<0.05) attenuated by clopidogrel treatment.

### Platelet inhibition protects the elastic and vascular smooth muscle cell lamina in aorta but does not prevent AAA rupture

**Figure 3:**
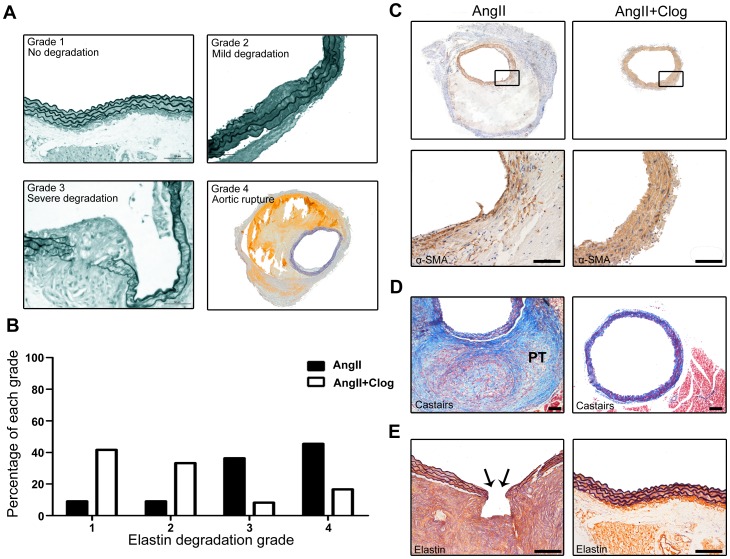
Platelet inhibition protects the elastic and vascular smooth muscle cell lamina in mouse aortas. **A**&**B**. Elastin degradation grading (4 grades) in Ang II-treated and Ang II+clopidogrel-treated mice. P<0.05. **C**. Representative immunostaining for α-smooth muscle actin (α-SMA) in aortic cross-sections. **D**. Aortas underwent Carstairs staining, in which platelets stain gray blue, fibrin red and collagen blue. Platelet-rich thrombi (PT) are highlighted in Ang II-treated rather than Ang II+clopidogrel-treated mice. **E**. Elastin van Gieson staining of aortic cross-sections of Ang II-treated and Ang II+clopidogrel-treated mice. Arrows display an area of complete elastic lamina rupture in Ang II-treated mice. Bar = 50 µm.

Elastin is the primary component of the media, constituting approximately 30% of the dry weight of aortas [24]. The elastic lamina is frequently disrupted and degraded in mice with AAA [25]. As shown in Figures 3A&B, clopidogrel treatment prevented elastin degradation after Ang II treatment for 4 weeks. Moreover, smooth muscle actin-positive area was preserved in aortas with clopidogrel treatment (Fig. 3C). These data suggest that protection of elastic and smooth muscle lamina is a major effect in suppression of AAA formation with clopidogrel treatment.

The most common complication of aortic aneurysms is acute rupture or dissection [26]. We analyzed aneurysm rupture by the presence of thrombus detected at the time of necropsy and by microscopy confirmation of elastin band rupture with associated dissection and organized formation in the aortic wall (Fig. 3D&E). When these microscopic elastin band ruptures evolve into a complete aortic wall rupture, the animal is going to die. Over the 4 weeks of Ang II infusion, 62.5% of the AAA mice infused with Ang II alone were found to have aneurysm rupture as compared with 66.7% of AAA mice receiving clopidogrel, and there was no significant difference in the incidence of aneurysm rupture between the 2 groups (Table 1).

### Platelet inhibition decreases macrophage infiltration into aneurysm tissue

**Figure 4:**
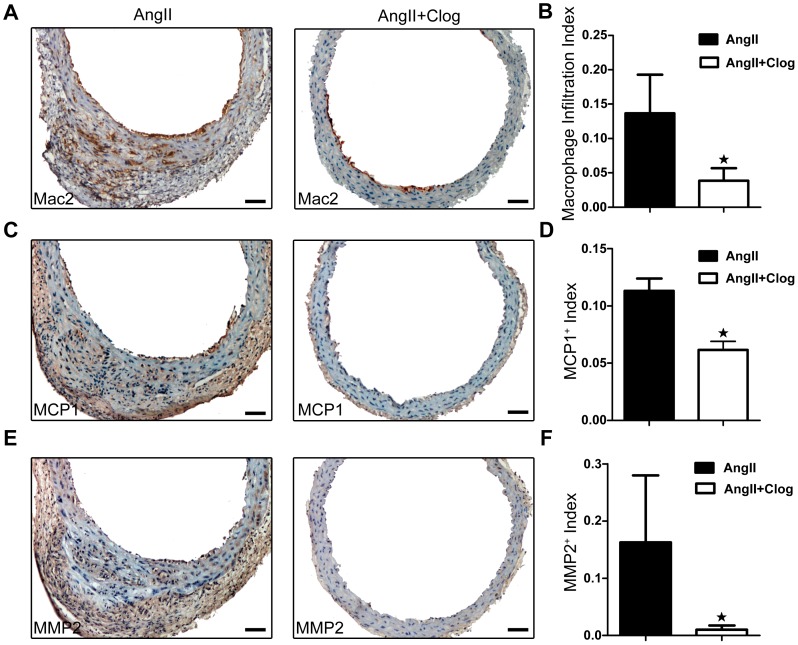
Platelet inhibition contributes to impaired inflammatory response and matrix metalloproteinase (MMP) expression in mouse aortas. **A**. Representative Mac-2 staining of suprarenal aortas form Ang II-treated and Ang II+clopidogrel-treated mice. **B**. Macrophage infiltration in abdominal aortas from Ang II-treated (n = 10) and Ang II+clopidogrel-treated (n = 10) mice. *P<0.05 compared with Ang II-treated mice only. **C**. Representative monocyte chemoattractant protein 1 (MCP-1) staining of suprarenal abdominal aortas from Ang II-treated and Ang II+clopidogrel-treated mice. **D**. MCP-1 expression in abdominal aortas from Ang II-treated (n = 10) and Ang II+clopidogrel-treated (n = 10) mice. *P<0.05 compared with Ang II-treated mice only. **E**. Representative immunostaining of MMP2 in suprarenal aortas from Ang II-treated and Ang II+clopidogrel-treated mice. **F**. Extent of MMP2 expression in abdominal aortae from Ang II-treated (n = 10) and Ang II+clopidogrel-treated (n = 10) mice. *P<0.05 compared with Ang II-treated mice only. All sections are shown with the lumen facing up. Bar = 50 µm.

Inflammation in the vasculature is a critical event for AAA formation, and infiltration of inflammatory cells plays a key role in the inflammatory process. To determine whether platelet inhibition affected vascular inflammation associated with AAA formation, we examined macrophages infiltration with serial tissue sections from mice prepared at the same distance from the point of maximal aortic diameter. As shown in Figures 4A&B, macrophages infiltration in AAA aortas, as assessed by Mac2^+^ cell number, was significantly reduced with clopidogrel treatment.

To elucidate the mechanism by which platelet inhibition participates in the inflammatory response, we analyzed the expression of MCP-1 in the aortic wall for its known role in macrophage recruitment and AAA formation [27], [28]. Clopidogrel treatment significantly decreased the expression of MCP-1 in the aortas of Ang II-treated mice (Fig. 4C&D). Thus, platelet inhibition suppressed the vascular inflammatory response in AAA.

### Platelet inhibition prevents Ang II-induced MMP activation and ROS production in aorta

**Figure 5:**
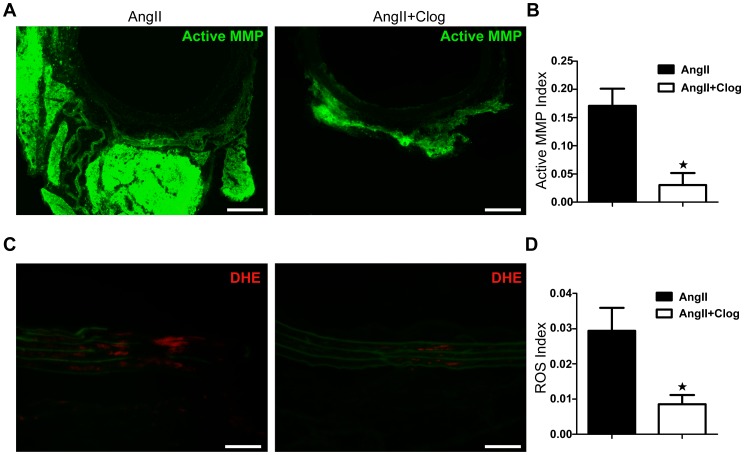
Platelet inhibition prevents MMP activity and reactive oxygen species (ROS) formation in mouse aortas. **A**. *In situ* zymography for gelatinase activity. Aortas from Ang II-treated and Ang II+clopidogrel-treated mice were analyzed. **B**. Densitometric analysis of MMP activity by DQ gelatin (a fluorogenic substrate used to detect protease activity) in aortas from Ang II-treated (n = 3) and Ang II+clopidogrel-treated (n = 3) mice. *P<0.05 compared with Ang II-treated mice only. **C**. *In situ* dihydroethidium (DHE) staining of aortas from Ang II-treated and Ang II+clopidogrel-treated mice. Green fluorescence in the media, observed in both groups, is due to elastin fiber autofluorescence. All sections are shown with the lumen at the top. **D**. Densitometric analysis of DHE fluorescence in aortas from Ang II-treated (n = 3) and Ang II+clopidogrel-treated (n = 3) mice. *P<0.05 compared with Ang II-treated mice only. Bar = 50 µm.

Macrophage-derived MMPs play a pivotal role in AAA development and aortic rupture [27], [29]. Because clopidogrel treatment significantly attenuated infiltration of macrophages, we hypothesized that the expression and activity of MMPs would be decreased with platelet inhibition. We then examined MMP-2 expression and MMP gelatinolytic activity in mice aortas with and without clopidogrel treatment. As shown in Figures 4E&F, MMP-2 expression in the aortic tissues of ApoE-deficient mice infused with Ang II was significantly reduced with clopidogrel treatment (P<0.05). Furthermore, *in situ* zymography showed significantly reduced MMP activity in the aortas of mice with clopidogrel treatment (Fig. 5A&B).

Oxidative stress could trigger activation of latent proforms of MMPs in aorta [30]. To further investigate the mechanisms by which platelet inhibition decreases MMP expression and activation, we performed *in situ* DHE staining for ROS production in aorta. As shown in Figures 5C&D, ROS production was significantly reduced in Ang II-treated mice aortas with clopidogrel treatment.

### Platelets activates macrophage MMPs

**Figure 6:**
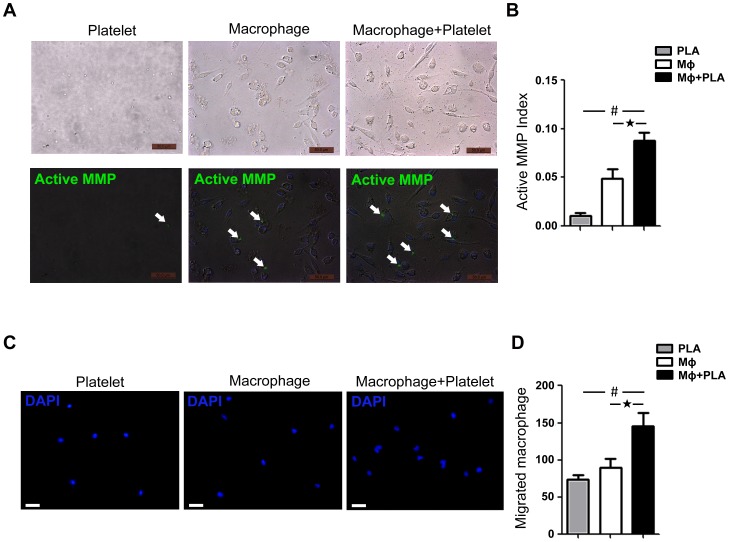
Platelet inhibition prevents mouse macrophage-derived MMP activity and macrophage migration *in vitro*. **A**. Colocalization of cells and activated MMPs. Cell nuclei were visualized by DAPI (blue) and activated MMPs were detected by DQ gelatin (green) (white arrows). **B**. Densitometric analysis of MMP activity by DQ gelatin in platelets cultured alone (n = 3), macrophages cultured alone (n = 3) and macrophages+platelets coculture (n = 3). *P<0.05 compared with macrophages cultured alone. ^#^P<0.05 compared with platelets cultured alone. **C**. Transwell assay of macrophages migration. Macrophages were placed on the upper chambers of transwell insets. The lower chamber was consisted of macrophage, platelets alone or macrophages+platelets coculture. Macrophages that had migrated to the lower chamber was counted by DAPI staining. **D**. Number of macrophages migrated to the lower chamber. *P<0.05 compared with macrophages cultured alone. ^#^P<0.05 compared with platelets cultured alone. Bar = 50 µm. PLA, platelet. Mφ, macrophage.

To demonstrate the role of platelets in regulation of MMP activity, macrophages and platelets were isolated and cocultured in a 1∶1 ratio, and MMP activity was assessed by *in situ* zymography. As shown in Figures 6A&B, macrophage MMP activity was significantly increased in the presence of platelets.

### Platelet is essential for macrophage migration

To determine the role of platelets in inflammatory cells infiltration, we performed a transwell migration assays. As shown in Figures 6C&D, coculture of platelets with macrophages increased macrophage migration significantly.

## Discussion

Inflammation plays a critical role in AAA formation. However, the early event initiating inflammation and AAA formation is still unknown. In this study, we found a large number of platelets and inflammatory cells in tissues of patients with AAA. Furthermore, treatment with clopidogrel, an inhibitor of platelets, in a murine model of AAA (ApoE-knockout mice infused with Ang II) significantly suppressed macrophage infiltration, ROS production and activation of MMPs, for a 47% decrease (P<0.05) in AAA formation. Interestingly, there was no significant difference in the incidence of aneurysm rupture with and without clopidogrel.

In an rat AAA model by chronic rejection of arterial allografts and xenografts, Dai et al found that blocking platelet activation with AZD6140 limited intraluminal thrombus biologic activities and impaired aneurysm development [11]. The result is obvious since platelets activation has been reported to be involved in xenograft immune rejection [31], [32], [33]. It is still unclear the roles platelets played in a natural AAA progression. Fouser reported intravascular hemolysis, disseminated intravascular coagulation, and a prolonged bleeding time in an AAA patient [15]. Bradbury et al performed a case record review of 65 AAA patients and found a direct correlation between platelet count on admission to the hospital and death after emergency repair of ruptured AAA [16]. Recent studies also demonstrate that anticoagulants might be responsible for a prolonged healing process and adverse clinical events including recurrence and rupture in the treatment of aortic dissection [12], [13], [14]. Given these inconsistent results, we investigated the effects of platelets inhibition on AAA initiation and progression and its mechanistic link using human AAA samples and the murine Ang II–infusion model of AAA.

The murine Ang II-infusion model of AAA share several biochemical and cellular similarities with human AAA [34]. Pathological features of this model include leukocyte infiltration, medial degeneration, and thrombus formation, all hallmarks of human AAA pathology [35]. These characteristics make it suitable for the study of platelets roles played in AAA initiation and progression. Clopidogrel, a platelet P2Y12 receptor inhibitor, has become an important therapeutic agent for patients with coronary heart disease [36]. It attenuates platelet activation and thus represents an effective pharmacological target for the inhibition of platelet aggregation [37].

Platelets and platelet-derived factors are long known to have a role beyond hemostasis in vascular diseases such as atherosclerosis and coronary artery disease [38]. Once vascular injury occurs, damaged vascular endothelial cells initiate an antifibrinolytic-coagulation cascade, which triggers the formation of blood clots [39]. Platelets accumulate within seconds to sites of vascular lesions and promote inflammation. Platelets are an important source of proinflammatory mediators and cytokines that can recruit inflammatory cells such as T lymphocytes and macrophages [32]. Previous studies found that vascular inflammation in the aortic wall has a pivotal role in AAA progression [7]. The recruitment of inflammatory cells accelerates AAA lesion development [7]. In agreement with these reports, we demonstrated a positive association of deposition of platelets and infiltration of inflammatory cells both in histological study of human AAA samples and in mouse experiments (Fig. 1&3D, Fig. 4A&B). Furthermore, we found enhanced infiltration of macrophages and expression of MCP-1 in the aortas of Ang II-treated mice, but such changes were suppressed by the platelet inhibitor clopidogrel (Fig. 4A–D).

Infiltration of inflammatory cells contributes to local inflammation through the secretion of inflammatory mediators, including cytokines, chemokine, oxygen radicals, and MMPs [7]. The expression of MMPs and ROS is a key mechanism in the initiation, progression and complications of AAA [7]. We found decreased MMP and ROS production in the aortic wall of Ang II-treated mice with clopidogrel treatment (Fig. 4E&F, Fig. 5C&D). Our findings suggest an important contribution of platelets in augmenting MMP activity in macrophages (Fig. 5A&B, Fig. 6A&B). Furthermore, both VSMC and endothelial cells (ECs) of the aorta have been shown to release MMP upon activation [25], [40]. It is also reported that platelet-derived chemokines regulate the MMP expression of VSMC and ECs [41], [42]. Therefore, our observed results of that clopidogrel protection of AAA could also be resulted from platelet regulation of VSMC and ECs.

Taken together, our results suggest that platelets contribute to the pathogenesis of AAA by activating MMP macrophages along with infiltration of inflammatory cells in the aortic wall. Platelets and their interaction with inflammatory cells are essentially involved in the initiation and progression of AAA. We characterized four pathological mechanisms by which platelet activation promotes AAA formation. First, platelet activation stimulates the recruitment of macrophages, contributes to ROS production synergistically with Ang II in aortas, and promotes MMP activity by inducing macrophage infiltration and augmenting ROS generation, and finally, activated MMPs induce the aortic degradation and rupture in mice.

The identification of platelets as a mediator of tissue damage associated with inflammation provides insight into the mechanism underlying a therapeutic intervention. The main AAA management approaches have been open surgical repair and endovascular stent-grafting [36]. However, the procedure is associated with significant operative risks and complications, so further elucidating the mechanisms of AAA formation and exploring a therapeutic target for AAA disease is important.

However, although platelet inhibition led to a 47% decrease in AAA formation, we found no significant difference in the incidence of aneurysm rupture with and without clopidogrel. This observation may be due to the roles of platelets in hemostasis. AAA wall stress has a high sensitivity and specificity in AAA rupture risk assessment [43]. The peak wall stress was higher for ruptured than nonruptured or asymptomatic AAA [44], [45]. Aneurysm rupture occurs when the arterial wall is unable to resist the dilating force of arterial pressure. Intraluminal thrombus could significantly lower AAA wall stress, and the effect is stronger for thicker and stiffer thrombi [46], [47]. Blood platelets have been implicated in catalyzing the formation of stable blood clots via coagulation cascade. Clopidogrel treatment could significantly inhibit the roles platelets play in the inflammatory process and in thrombosis. As we found, platelet inhibition led to a decrease in AAA formation by reducing the vascular inflammatory response.

In summary, this study provides evidence of the involvement of platelets in concert with other inflammatory cells and suggests a key role for platelet activation in AAA formation and other cardiovascular diseases associated with inflammation.
